# Retraction: Zinc sulfate contributes to promote telomere length extension via increasing telomerase gene expression, telomerase activity and change in the *TERT* gene promoter CpG island methylation status of human adipose-derived mesenchymal stem cells

**DOI:** 10.1371/journal.pone.0292985

**Published:** 2023-10-26

**Authors:** The PLOS ONE Editors

Following the publication of this article [1], concerns were raised regarding results presented in Figs 2, 3, and 4. Specifically,

- The Fig 2D panel of this article [1] appears similar to the Figure 1 CD56 panel of [2] despite representing results from different experiments on different tissues by different research groups.
- The Fig 3 top left and bottom left FACS panels appear more similar than expected from independent results.
- In the Fig 4H NGF panel there appear to be irregularities suggestive of splice lines.

During the assessment of the above concerns, PLOS noted that the primary data were not provided with the published article [1], contrary to the Data Availability statement. Upon editorial request, the corresponding author provided underlying data for their study. Assessment of the individual level data underlying the graphs published in this article raised additional concerns regarding the reliability and integrity of these data.

Regarding Fig 2D, the corresponding author stated that of the panel is correctly reported in [1] and that the duplication is due to an error in [2]; this is supported by the presentation of the same results in an MSc thesis [3]. The corresponding author also noted that an author of [1] and Ali Jahanbazi Jahan-Abad, an author of [2,3], studied related topics under the same supervisor at Tarbiat Modares University. The contributions of Dr. Jahan-Abad were not acknowledged in [1] even though results from their MSc thesis were included in the article [1].

Regarding the concerns with the FACS results, the corresponding author stated that the results presented in Fig 3 were obtained from cells grown in the same flask before being subjected to treatment with different antibodies. The corresponding author states that the FACS results are expected to be similar because the cells were obtained from the same population. The *PLOS ONE* editors remain concerned that the FACS panels appear more similar than would be expected from technical repeat samples. The underlying FACS data provided by the authors suggested there may have been an error during the preparation of Fig 3 in [1], but did not fully resolve the Editorial concerns with the published FACS results.

The corresponding author commented that the irregularities in the Fig 4H NGF panel are the result of a technical defect with the imaging device. In absence of the original gel data underlying the published results this issue cannot be resolved.

In light of the above concerns that question the reliability and integrity of the published results, the *PLOS ONE* Editors retract this article. At the time of retraction, this article was republished to update the copyright statement for the Fig 2D panel.

RF, EF, and SAMN did not agree with the retraction and stand by the article’s findings. NZ either did not respond directly or could not be reached.
